# Graded expression of microRNA-371a-3p in tumor tissues, contralateral testes, and in serum of patients with testicular germ cell tumor

**DOI:** 10.18632/oncotarget.27565

**Published:** 2020-04-21

**Authors:** Gazanfer Belge, Finja Hennig, Cansu Dumlupinar, Francesca Grobelny, Klaus Junker, Arlo Radtke, Klaus-Peter Dieckmann

**Affiliations:** ^1^ Faculty of Biology and Chemistry, University of Bremen, Bremen, Germany; ^2^ Department of Pathology, Klinikum Bremen-Mitte, Bremen, Germany; ^3^ Department of Urology, Asklepios Klinik Altona, Hamburg, Germany

**Keywords:** testicular germ cell tumors, microRNA-371a-3p, serum, contralateral testes, *in situ* hybridization

## Abstract

**Background:** Serum levels of microRNA-371a-3p represent a specific tumor marker of testicular germ cell tumors (GCTs) but the origin of circulating miR-371a-3p is not finally resolved. The correlation between miR-levels in tissue and serum is unclear.

**Results:** MiR-levels in GCT tissue are 399-fold higher than in contralateral testicular tissue and 5843-fold higher than in non-testicular tissue. MiR tissue levels correlate with corresponding serum levels (r^**2**^ = 0.181). ISH detected miR-371a-3p intracellularly in GCT cells except teratoma. A low expression was also detected in normal testicular germ cells.

**Conclusions:** Circulating miR-371a-3p is specifically derived from GCT tissue. The miR is present in GCT cells except teratoma. A low expression is also found in normal testicular tissue but not in non-testicular tissue. MiR-371a-3p levels in tissue and serum correlate significantly. This study underscores the usefulness of serum miR-371a-3p as tumor marker of GCT.

**Patients and methods:** Expression levels of miR-371a-3p were concurrently measured in tissues of GCT, contralateral testes (***n*** = 38), and in serum (***n*** = 36) with real time PCR. For control, 5 healthy testicles and 4 non-testicular tissue samples were examined. MiR-levels were compared using descriptive statistical methods. We also performed *in situ* hybridization (ISH) of GCT tissue with a probe specific for miR-371a-3p.

## INTRODUCTION

Serum levels of microRNAs (miRs) of the clusters miR-371-373 and miR-302/367 have been suggested as novel biomarkers of testicular germ cell tumors (GCTs) [1–3]. Of the candidate miRs, miR-371a-3p appears to be the most promising serum marker of GCT with a sensitivity of 90.1% and specificity of 94.1% [4, 5] outperforming the classical markers (alpha fetoprotein, beta human chorionic gonadotropin, lactate dehydrogenase) with their sensitivities of less than 50% [6]. Apparently, miR-371a-3p features almost all of the qualities a valuable tumor marker is supposed to have [7] since it correlates with clinical stages, and tumor sizes, it highlights response (or non-response) to therapy, and it is present in cases with relapsing GCT suggesting a prominent role of this miR upon follow-up examinations [8–15]. Preliminary data also suggest a possible role of the test upon evaluation of residual masses after chemotherapy [13, 16, 17].

While lots of clinical data suggest a strong correlation between tumor burden and miR-371a-3p serum expression, only limited evidence is available to show that serum-based miRs do primarily originate from GCT cells and do not represent any unspecific side reaction of the testis to invasive GCT.

Testicular vein blood sampling had demonstrated that the tumor-bearing testis is most likely the source of circulating miR-371a-3p [18].

Early experiments had provided evidence for the presence of miRs 372-373 in GCT tissue [19]. Later, high-throughput screening and microarray expression profiling documented miRs 371-373 to be present in tissue of GCTs [20–24]. A study using RNA extraction from formalin-fixed paraffin embedded GCT tissue again demonstrated the presence of miR-371a-3p in tumor tissue with different expression levels in the various histological subtypes of GCT [25]. All of these studies did not directly compare the miR-expression levels in tumor tissue with corresponding serum levels in the individual patients. The only study to date that evaluated both tissue expression levels and corresponding serum levels did not find a clear correlation between these levels [26].

The aim of the present study was to further clarify the origin of circulating miR-371a-3p by measuring this miR in serum of patients with GCT and concurrently in tissues of the tumor and of the contralateral testes in the same patients. The second goal was to explore if increasing levels of miR371 in GCT tissue would translate into higher serum levels of the miR.

## RESULTS

### Results of microRNA expression investigations in tissues

**Figure 1:**
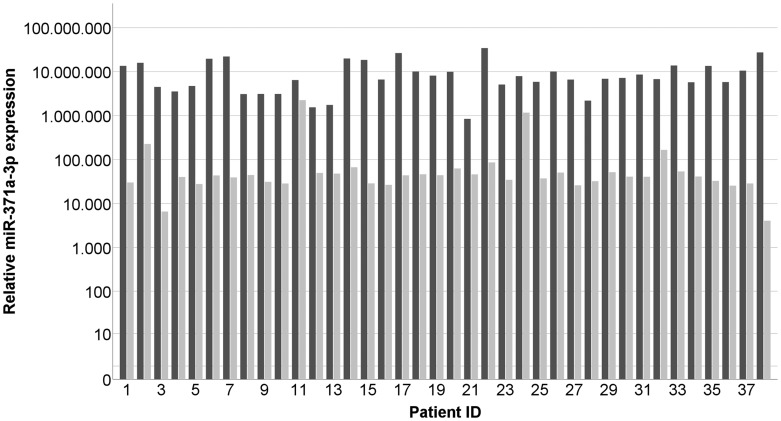
Individual results of measuring miR-371a-3p expressions in GCT tumor tissue samples (dark grey) and the corresponding contralateral testicle (light grey). *n* = 38. The patient ID is identical with data sets in Table 1. The y-axis is displayed in a logarithmic scale.

**Figure 2:**
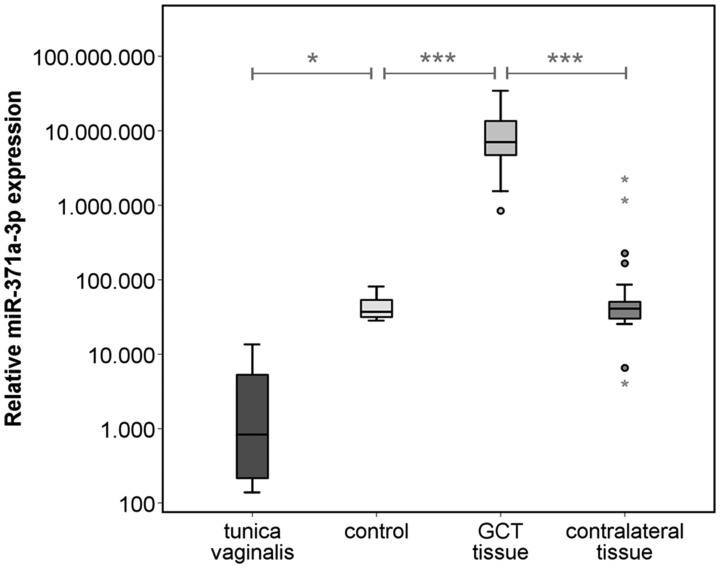
Relative miR-371a-3p expression in GCT tissue (*n* = 38) and corresponding contralateral testicular tissue (*n* = 38) of the same patients, healthy controls (*n* = 5) and non-testicular tissue samples of the tunica vaginalis (*n* = 4). The y-axis is plotted in a logarithmic scale. ^***^ *p* < 0.001.

The median miR-371a-3p expressions in tumor, corresponding contralateral testicular tissue, testicular tissue of healthy controls, and non-testicular tissue of testis-surrounding tunica vaginalis were RQ = 7,040,480.1 (IQR 4,713,672.2–13,518,390.0), RQ = 40,974.1 (IQR 30,119.7–50,549.9), RQ = 37,081.7 (IQR 31,617.2–53,543.4), and RQ = 1,204.9 (IQR = 237.5–7,809.9) respectively. Thus, the individual miR-371a-3p levels found in tumor tissue are on average 399-fold higher than those of the corresponding contralateral testicular tissue (*p* < 0.001) (Figures 1, 2). Likewise, expression levels are significantly higher in GCT tissue than in healthy testicular tissue (*p* < 0.001). MiR-371a-3p expression in healthy testicular tissue is not significantly different from that in contralateral testicular tissue (*p =* 0.985). The miR-371a-3p expression in non-testicular tissue (tunica vaginalis) showed 30.8-times lower values than testicular tissue of healthy controls (*p* < 0.05) (Figure 2). In addition, one sample of epididymis was analyzed with the lowest miR-371a-3p expression of all tissue samples (RQ = 42.03) (Table 1). There is no difference detectable between the miR-371a-3p expression of seminomas and nonseminomas (*p =* 0.941). Likewise, there is no difference between miR-371a-3p expressions in tissues of CS1 and CS 2/3 cases (*p =* 0.262) (Supplementary Figures 1 and 2).

**Table 1:** Clinical data of analyzed patients

Patient ID	Age [yrs]	Diameter [mm]	Histology	Clinical Stage	site	RQ miR-371a-3p
1	38	46	Seminoma	CS2	Contralateral tissue	30,119.70
					GCT tissue	13,518,389.95
					Serum	2,881.17
2	34	36	Seminoma	CS1	Contralateral tissue	226,385.18
					GCT tissue	15,854,840.19
					Serum	5,509.17
3	38	19	Seminoma	CS1	Contralateral tissue	6,555.18
					GCT tissue	4,490,423.24
					Serum	669.09
4	28	31	Non-Seminoma	CS2	Contralateral tissue	40,019.62
					GCT tissue	3,547,619.87
					Serum	6,537.03
5	29	24	Non-Seminoma	CS1	Contralateral tissue	27,715.78
					GCT tissue	4,713,672.19
					Serum	3,287.19
6	36	54	Seminoma	CS1	Contralateral tissue	43,490.72
					GCT tissue	19,655,367.47
					Serum	39,571.18
7	32	42	Seminoma	CS1	Contralateral tissue	39,196.03
					GCT tissue	22,113,437.94
					Serum	41,500.32
8	51	18	Seminoma	CS1	Contralateral tissue	44,404.55
					GCT tissue	3,088,382.47
					Serum	1,096.01
9	45	67	Seminoma	CS1	Contralateral tissue	31,181.87
					GCT tissue	3,109,863.87
					Serum	7,068.64
10	41	45	Seminoma	CS1	Contralateral tissue	28,494.98
					GCT tissue	3,109,863.87
					Serum	6,313.85
11	25	26	Seminoma	CS1	Contralateral tissue	2,245,211.62
					GCT tissue	6,439,065.97
12	36	31	Seminoma	CS1	Contralateral tissue	49,612.64
					GCT tissue	1,544,191.24
					Serum	2,131.85
13	40	37	Seminoma	CS1	Contralateral tissue	47,922.65
					GCT tissue	1,749,389.37
					Serum	10,019.87
14	27	41	Non-Seminoma	CS1	Contralateral tissue	66,839.81
					GCT tissue	19,929,746.17
					Serum	120,356.55
15	36	33	Non-Seminoma	CS1	Contralateral tissue	28,693.18
					GCT tissue	18,466,664.88
					Serum	4,629.83
16	37	52	Non-Seminoma	CS1	Contralateral tissue	26,586.75
					GCT tissue	6,620,092.75
					Serum	40,760.69
17	30	22	Seminoma	CS2	Contralateral tissue	43,793.22
					GCT tissue	26,480,371.01
					Serum	2,652.53
18	34	73	Seminoma	CS3	Contralateral tissue	46,290.23
					GCT tissue	10,103,977.53
					Serum	56,414.42
19	31	68	Seminoma	CS1	Contralateral tissue	44,036.74
					GCT tissue	8,110,840.54
					Serum	4,772.14
20	49	18	Seminoma	CS1	Contralateral tissue	62,623.65
					GCT tissue	9,930,397.24
					Serum	288.5
21	47	30	Seminoma	CS1	Contralateral tissue	46,002.35
					GCT tissue	841,392.83
					Serum	100.78
22	34	34	Seminoma	CS1	Contralateral tissue	85,902.95
					GCT tissue	34,412,276.52
					Serum	16,913.42
23	34	33	Seminoma	CS1	Contralateral tissue	34,646.45
					GCT tissue	5,083,603.22
					Serum	469.32
24	38	50	Non-Seminoma	CS3	Contralateral tissue	1,167,847.12
					GCT tissue	7,899,993.31
					Serum	4,434.08
25	34	11	Seminoma	CS1	Contralateral tissue	37,210.44
					GCT tissue	5,859,800.02
					Serum	290.67
26	35	75	Seminoma	CS2	Contralateral tissue	50,549.88
					GCT tissue	10,076,002.16
					Serum	3,402.60
27	31	n. a.	Non-Seminoma	CS2	Contralateral tissue	25,931.54
					GCT tissue	6,601,763.38
					Serum	32,461
28	41	6	Seminoma	CS1	Contralateral tissue	32,214.44
					GCT tissue	2,191,397.85
					Serum	445.85
29	66	29	Seminoma	CS2	Contralateral tissue	51,647.83
					GCT tissue	6,891,659.54
					Serum	2,007.83
30	53	42	Seminoma	CS1	Contralateral tissue	40,860.52
					GCT tissue	7,189,300.62
31	47	37	Seminoma	CS2	Contralateral tissue	40,550.16
					GCT tissue	8,585,198.14
					Serum	231.91
32	52	48	Seminoma	CS1	Contralateral tissue	166,068.62
					GCT tissue	6,777,961.45
					Serum	840.1
33	40	56	Seminoma	CS1	Contralateral tissue	53,692.02
					GCT tissue	13,792,876.25
					Serum	4,638.43
34	30	45	Non-Seminoma	CS2	Contralateral tissue	41,087.73
					GCT tissue	5,743,186.58
					Serum	11,405.33
35	47	14	Non-Seminoma	CS1	Contralateral tissue	32,709.45
					GCT tissue	13,499,662.47
					Serum	516.07
36	34	47	Seminoma	CS1	Contralateral tissue	25,468.39
					GCT tissue	5,799,190.10
					Serum	9,597.35
37	51	22	Seminoma	CS1	Contralateral tissue	28,593.90
					GCT tissue	10,547,662.66
					Serum	3,634.19
38	47	16	Seminoma	CS2	Contralateral tissue	4,060.44
					GCT tissue	27,376,221.46
					Serum	18,247.61
39	47	0	Control	normal	Control	31,617.16
40	63	0	Control	normal	Control	53,543.36
41	19	0	Control	normal	Control	37,081.71
42	60	0	Control	normal	Control	28,298.15
43	54	0	Control	normal	Control	81,156.55
44	78	0	Control	normal	*Tunica vaginalis*	138.88
45	20	0	Control	normal	*Tunica vaginalis*	336.12
46	52	0	Control	normal	*Tunica vaginalis*	42.03
47	33	0	Control	normal	*Tunica vaginalis*	13,546.11
48	46	0	Control	normal	Epididymis	2,073.66

Abbreviations: CS: Clinical stage, GCT: Germ cell tumor, mm: millimeter, RQ: Relative quantity, yrs: Years.

The ROC analysis based on miR measurements of GCT tissue and the corresponding contralateral tissue in 38 patients revealed an area under the curve of 0.997. GCT tissue can thus be discriminated from the corresponding contralateral tissue with a diagnostic sensitivity of 100% and a specificity of 94.7% (Supplementary Figure 3).

### Comparison of miR-371a-3p expression in tissue with serum levels

**Figure 3:**
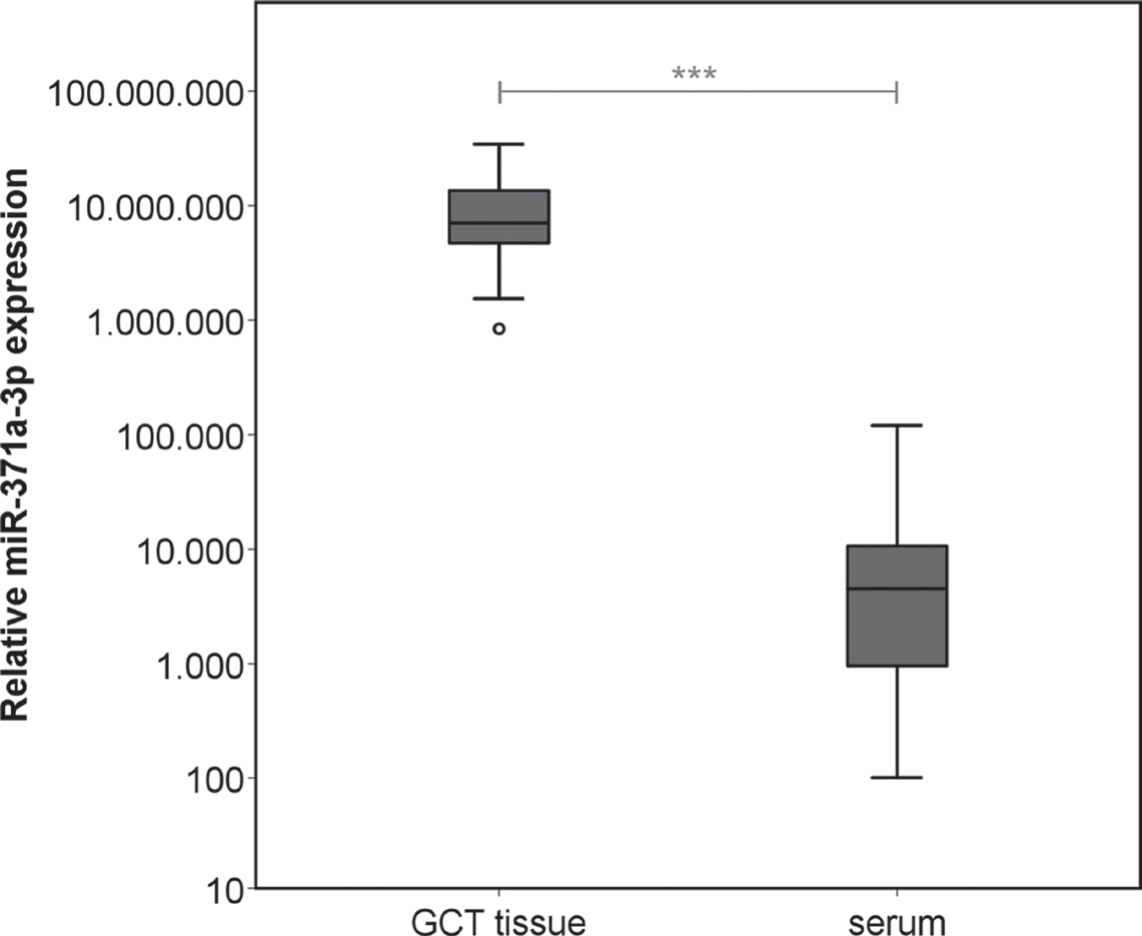
Relative miR-371a-3p expression in GCT tissue (*n* = 38) and corresponding preoperative serum samples of the same patients (*n* = 36). The y-axis is plotted in a logarithmic scale. ^***^ *p* < 0.001.

**Figure 4:**
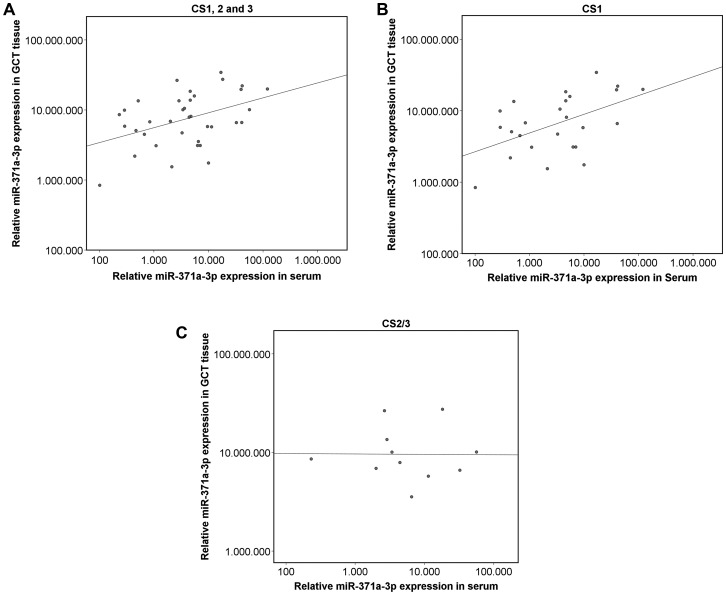
Scatterplot of the relative miR-371a-3p expression in GCT tissue and corresponding serum levels. The axes in all parts are depicted in a logarithmic scale. (**A**) Entire GCT cohort (*n* = 36) (*p* ≤ 0.05) (r^2^ = 0.181). (**B**) Only CS1 patients (*n* = 25; *p* ≤ 0.05) (r^2^ = 0.257). (**C**) Only CS2/3 patients (*n* = 11; *p* ≥ 0.05) (r^2^ = 9.5 × 10^−5^).

MiR-371a-3p expression levels in GCT tissue are significantly higher than corresponding serum levels (*p* < 0.001) (Figure 3). There is a significant positive correlation between tissue levels and those found in serum (*p* < 0.05) (r^2^ = 0.181) (Figure 4A). The correlation is stronger in the sub cohort of CS1 patients (*p* < 0.05) (r^2^ = 0.257) (Figure 4B) than in cases with CS2 and CS3 where it is not significant (*p* > 0.05) (r^2^ = 9.5 × 10^–5^) (Figure 4C).

### Results of *in situ* hybridization

**Figure 5:**
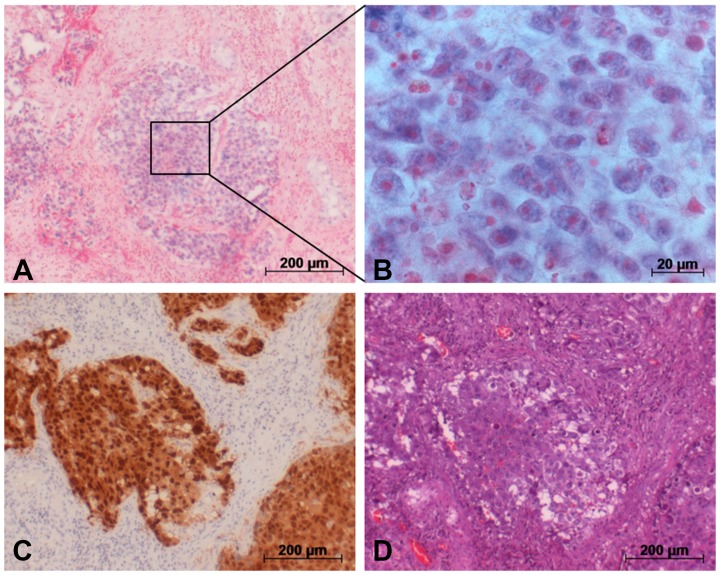
Detection of miR-371a-3p in GCT tumor (EC) via *in situ* hybridization. (**A**) *In situ* hybridization with a probe against miR-371a-3p causes blue staining in cells. (**B**) Section from A. (**C**) Immunohistochemical staining of the same area with an OCT4 antibody for identification of EC cells. (**D**) H&E staining of the same area.

**Figure 6:**
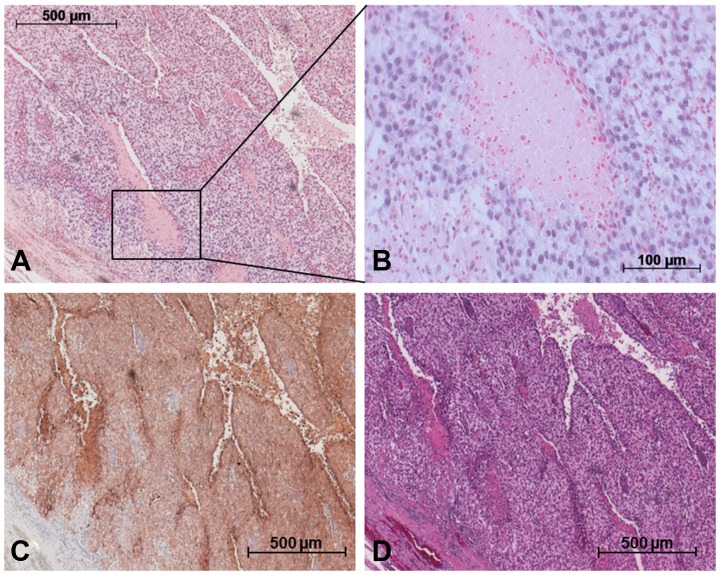
Detection of miR-371a-3p in GCT mixed tumor including SE and YST via *in situ* hybridization. (**A**) *In situ* hybridization with a probe against miR-371a-3p causes blue staining in cells. (**B**) Section from A. (**C**) Immunohistochemical staining of the same area with PLAP antibody for identification of SE cells. (**D**) H&E staining of the same area.

Figures 5 and 6 show the ISH results in the various GCT subtypes. The blue stain highlights cells expressing miR-371a-3p intracellularly (Figures 5A, 5B, 6A and 6B). An expression of miR-371a-3p was found in all subtypes of GCT except teratoma (Supplementary Figure 4). Identification of the different subtypes was achieved by additional staining with OCT4 for EC (Figure 5C), PLAP for seminoma (Figure 6C) and Glypican 3 for YST (Supplementary Figure 4). In contralateral tissue only isolated germ cells showed blue ISH signals.

## DISCUSSION

There are five main results of this study: (1) The miR-371a-3p expression levels are markedly higher in GCT tissue than in the tissue of the contralateral testis and in normal testes, while non-testicular tissue from testis-surrounding layers (tunica vaginalis) has even much lower expression. (2) There is clear evidence from ISH studies that miR-371a-3p is localized within GCT tumor cells. (3) The miR expression level in GCT tissue is much higher than in serum. (4) In patients with localized disease (CS1), the GCT tissue miR-levels significantly correlate with the corresponding serum levels. (5) There is a baseline miR-371a-3p expression in tissues of contralateral testes of GCT patients and also in normal testes.

### MiR-371a-3p levels in the tissues of tumor and contralateral testis

A pair-wise comparison of the expression of miR-371a-3p in GCT tissue with corresponding contralateral testicular tissue is a unique opportunity to look for the cellular origin of circulating miR-371a-3p molecules. The miR-level in GCT tissue is 399-fold higher than in contralateral testis tissue and the difference between GCT tissue and testicular tissue of control patients is almost the same. This result clearly points to the GCT tissue as the source of circulating miR-371a-3p. Previous reports had already pointed to the presence of the miR-371-373 cluster in GCT tissue, without quantitative measurements [20], [21]. Our result is consistent with data from measuring the miR in testicular vein blood where a 195-fold higher level was found than in systemic circulation [18]. The present finding of a base-line expression of miR-371a-3p in contralateral testis tissue and in healthy testicular tissue mirrors the finding of a 4-fold higher miR-expression in testicular vein blood compared to peripheral blood in healthy males [18]. These data suggest the presence of this particular miR even in normal testicular tissue though in low quantity and not exclusively in GCT tissue. This assumption is supported by the findings of Boellaard *et al.* who recently documented the presence of miR-371a-3p in normal testicular tissue and its absence in other parts of the urogenital tract [27]. As miR-371 has been shown to be specifically associated with human stem cells [28–30] and as it is found in seminal plasma, too [31, 32], it is rational to assume that this miR is specifically generated by normal testicular germ cells and most probably even more by the cells of GCT.

In serum, patients with nonseminomas showed significantly higher miR-371a-3p expression, and CS2/3 patients had higher levels than those with CS1 [5]. In tissue no difference was detectable between the miR-371a-3p expression of seminomas and nonseminomas nor between tissues of CS1 and CS 2/3 cases. Yet, these results should be interpreted with caution as only a small patient collective was examined.

### Intracellular localization of miR-371a-3p by ISH

*In situ* hybridization clearly demonstrated microRNA-371-3p to be localized intracellularly in the cells of GCT. Thus, the ISH experiments morphologically supplement the data obtained by measuring the miR in homogenized GCT tissue.

In conjunction with previous reports on the presence of the miR-371-373 cluster in GCT tissue [20, 21, 25, 33] there is now ample evidence for the origin of circulating miR-371a-3p from testicular tumor cells.

Of note, all subtypes of GCT except teratoma stained positive for the miR-371a-3p probe. The absence of miR-371a-p staining in teratoma cells is consistent with the non-expression of this miR in serum of GCT patients [4, 5, 16]. Likewise, in an evaluation of miR-371a-3p expression levels in GCT subtypes by RNA extraction from formalin fixed paraffin embedded orchiectomy specimens, Vilela-Salgueiro *et al.* revealed a strong expression in all GCT subtypes except teratoma [25]. The reason for the non-expression of the miR by teratoma is probably related to the analogies of GCT biology and the human embryonal development [34, 35]. While most of the GCT subtypes mimic early developmental stages of embryonal development and accordingly retain their biochemical characteristics including the microRNA profile of stem cells, the teratoma subtype represents a more advanced and more mature histological subtype that has lost all of the biochemical characteristics of stem cells particularly the typical expression of miR-371a-3p [36].

### Correlation of miR-expression levels in tissue with serum levels

Systematic paired measurements of miR-expression levels in GCT tissue and corresponding serum levels had not been reported so far. One early pilot study had indicated that miR-expression levels of tissue and serum do not correlate [24]. However, the present systematic analysis of 36 patients revealed a significant correlation of the expression levels of the two compartments. It is of note that this correlation was not significant in cases with clinical stages CS2 and 3. As in advanced clinical stages the marker substance is released from both the primary tumor and metastatic seeds it is rational that the correlation of tissue expression with serum levels is only significant in cases confined to the testis. The serum level of circulating miR-371a-3p is obviously a product of the number of miR-producing tumor cells (tumor bulk) as shown previously [5] and of the specific secreting capacity of the individual GCT as shown herein. Most probably, additional biological determinants e. g. direct vascular invasion of the tumor and other hitherto unknown factors do also affect the serum level of miR-371a-3p.

In all, the present evaluation confirms the understanding that circulating miR-371a-3p-are specifically derived from cells of testicular germ cell neoplasms.

This miR, thus represents a specific tumor marker for GCTs, which is not expressed in other diseases. By contrast, the specificity of the classical tumor marker AFP is considerably hampered by its association with non-GCT related conditions, such as liver diseases [37].

## MATERIALS AND METHODS

### Patients for tissue and serum investigations

Preoperative serum samples and corresponding tissue specimens were collected from patients (median age 36.5) undergoing surgery for testicular tumor. GCT tissue was taken from 38 orchiectomy specimens, and contralateral testis tissue was taken from corresponding contralateral biopsy specimens. This surgical procedure was routinely performed on all patients with suspected testis tumor to look for Germ cell neoplasia *in situ* (GCNis) according to institutional guide-lines. None of the patients enrolled in this study had contralateral GCNis. All tissue specimens were kept frozen at –80° C under further processing. Histologically, the GCT tissue specimens consisted of 29 seminomas and nine nonseminomas. Clinically, 27 patients had clinical stage 1 (CS1) and eleven CS 2 and 3 (CS2/3). Preoperative serum samples were obtained from 36 of the 38 patients. For control, testicular tissue was obtained from five men without GCT undergoing orchiectomy for epididymitis. Furthermore, four samples of testis surrounding tunica vaginalis and one specimen of the epididymis were analyzed. All patients had given informed consent prior to surgery. Ethical approval of the study was provided by Ärztekammer Bremen (reference No 301, 2011). All study activities had been conducted according to the Declaration of Helsinki of the World Medical Association (as amended by the 64th General Assembly, 2013). Individual data of the patients and controls are listed in Table 1.

### Patients for histological investigation of presence of microRNAs in GCT cells

Formalin-fixed paraffin-embedded (FFPE) samples of six patients with testicular GCTs were analyzed by immunohistochemistry and *in situ* hybridization (ISH) to look for the presence of miR-371a-3p in tumor tissue. Histologically, the GCTs comprised of three mixed nonseminomatous tumors (embryonal carcinoma, yolk sac tumor, and chorio carcinoma), one pure seminoma, one pure embryonal carcinoma and one teratoma. In addition, one tissue specimen of a contralateral testis was analyzed.

### Extraction and measurement of miRNAs

Tumor and contralateral testis tissue (10–50 mg) was homogenized in 1000 μL TRIzol^®^ Reagent following the manufacturer’s instructions (Fisher Scientific, Schwerte, Germany) using a TissueLyser (Qiagen, Hilden, Germany) with 5 mm steel beads for 10 min at 30 Hz. The extracted RNA was resuspended in 50 μl nuclease-free water.

For the measurement of miR-371a-3p levels, RNA was isolated from 200 μL Serum using the miRNeasy mini Kit (Qiagen, Hilden, Germany) according to the manufacturer’s description. Reverse transcription (RT) was performed with 10–20 ng/μL RNA isolated from tissue and 6 μL RNA isolated from Serum, using the TaqMan MicroRNA Reverse Transcription Kit (Applied Biosystems, Darmstadt, Germany). Standard PCR was carried out for preamplification of the cDNA with TaqMan Assays for miR-371a-3p (assay ID 002124) and the endogenous control miR-93-5p (assay ID 000432) in a 1:100 dilution. Measurement of the miRNA expression was performed with quantitative real-time PCR (RT-qPCR) on a 7500 Fast Real-Time PCR System (Applied Biosystems, Darmstadt, Germany) using FAST Start Universal Probe Master (Roche Diagnostics, Mannheim, Germany) and the undiluted TaqMan Assays. The relative quantity (RQ) was calculated using the 2^-ΔΔCT^-method [38].

### Immunohistochemistry

For morphological identification of the histological type of GCTs sections of 5 μm of FFPE-blocks were analyzed with hematoxylin and eosin stain (H&E stain), OCT4, PLAP and glypican 3. Staining with hematoxylin and eosin with standard histological techniques were used to distinguish between tumor-free areas and tumor tissue. OCT4 staining for identification of embryonal carcinomas (EC), PLAP staining for seminomas (SE) and glypican 3 for yolk-sac tumors (YST) (Diagnostic BioSystems, Pleasanton, CA, USA) were then conducted according to institutional standard operating procedures [39, 40].

### MicroRNA *in situ* hybridization

After immunohistochemical identification of the particular GCT-subtypes, the corresponding tumor sections were subsequently processed for *in situ* hybridization (ISH) with a miRCURY LNA probe (Exiqon, Vedbaek, Denmark; probe ID 38555-15) specific for miR-371a-3p. The protocol was performed according to the manufacturer’s instructions using a proteinase-K concentration of 15 μg/ml, a hybridization temperature of 51° C and a probe concentration of 80 nM. Microscopic evaluations were performed on an Axioskop 2 plus microscope (Zeiss, Göttingen, Germany). Histological findings were documented using the AxioCam HRc digital camera (Zeiss, Göttingen, Germany) and then edited with AxioVision Software v.4.8 (Zeiss, Göttingen, Germany). Presence of miR-371a-3p within GCTs was defined by distinct blue staining of the cells, and accordingly, only these cells were considered miR-371a-3p positive. Only the presence or absence of the miR-371a-3p in the specimen was evaluated, no quantification was attempted.

### Statistical methods

The Wilcoxon signed rank test was used for comparison of dependent subgroups. The Mann-Whitney U test was used to compare median miRNA expressions among the various subgroups. Receiver Operating characteristics (ROC) curves were calculated to analyze the sensitivity and specificity of tissue miR-levels to distinguish GCT tissue from non-tumorous tissue. Spearman’s rank correlation coefficient was calculated to determine correlations. All tests were two-sided and significance was assumed at *p* < 0.05. Statistical analysis was performed using SPSS version 24 (IBM, Armonk, NY, USA).

## LIMITATIONS

The results of the present study rest on 36 patients only, thus the statistical power is still limited. With respect to sample processing, the time for transfer of the tissue specimens from operation site to the laboratory i. e. the time-interval until conservation in the freezer (–80° C) varied from 12 hours to 36 hours depending on the conditions of surface mail. Thus, deterioration of some samples during transfer cannot entirely be excluded. We could not correlate the miR-measurements in contralateral testicular tissue specimens with the quality of spermatogenesis because these data were not available.

## CONCLUSIONS

The present study provides much of evidence for the understanding that circulating miR-371a-3p molecules are specifically derived from testicular GCT cells. The inability of teratoma cells to produce miR-371a-3p is confirmed on the tissue level. There is a significant correlation of the miR-expression levels in GCT tissue with corresponding serum levels. Normal testicular tissue displays a low baseline expression of miR-371a-3p pointing to the role of the miR in human stem cells.

## SUPPLEMENTARY MATERIALS



## Abbreviations

AFP: Alpha fetoprotein
bHCG: Beta human chorionic gonadotropin
CS: Clinical stage
EC: Embryonal carcinomas
FFPE: Formalin-fixed paraffin-embedded
GCNis: Germ cell neoplasia *in situ*
GCT: Germ cell tumor
H&E: Hematoxylin and eosin
IQR: Inter quartile range
ISH: *In situ* hybridization
LDH: Lactate dehydrogenase
miR: micro RNA
M371: miR-371a-3p
OCT4: Octamer-binding transcription factor 4
PLAP: Placental alkaline phosphatase
qPCR: Quantitative real-time PCR
RT: Reverse transcription
ROC: Receiver operating characteristics
RQ: Relative quantity
SE: Seminoma
SPSS: Statistical package for the social sciences
YST: Yolk-sac tumor
