# Supplementary material for: Graded expression of microRNA-371a-3p in tumor tissues, contralateral testes, and in serum of patients with testicular germ cell tumor
Source: Oncotarget. 2020 Apr 21;11(16):1462–73. doi: 10.18632/oncotarget.27565 (PMC7185068; doi:10.18632/oncotarget.27565)
Supplement: Supplementary file 1 [file oncotarget-11-1462-s001.pdf]

Graded expression of microRNA-371a-3p in tumor tissues, contralateral testes, and in serum of patients with testicular germ cell tumor

SUPPLEMENTARY MATERIALS

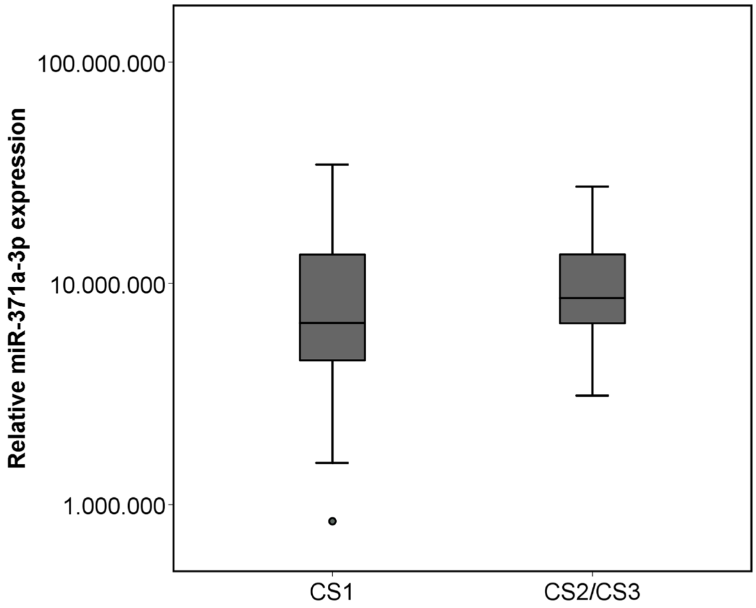

Supplementary Figure 1: Comparison of the relative miR-371a-3p expression in primary tumor tissue of patients with CS1 (*n* = 27) and with CS2/3 (*n* = 11); *p* = 0.262.

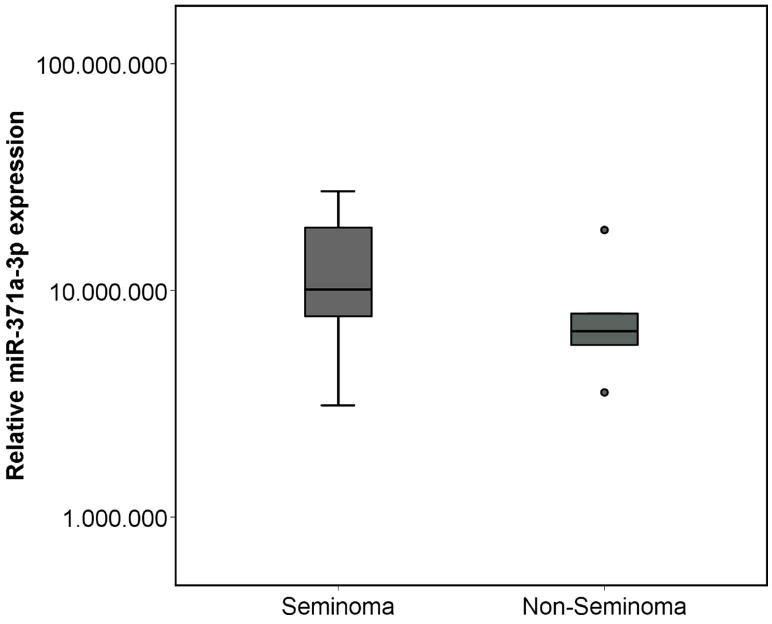

Supplementary Figure 2: Comparison of the relative miR-371a-3p expression in seminoma (*n* = 29) and nonseminoma (*n* = 9). *p* = 0.941.

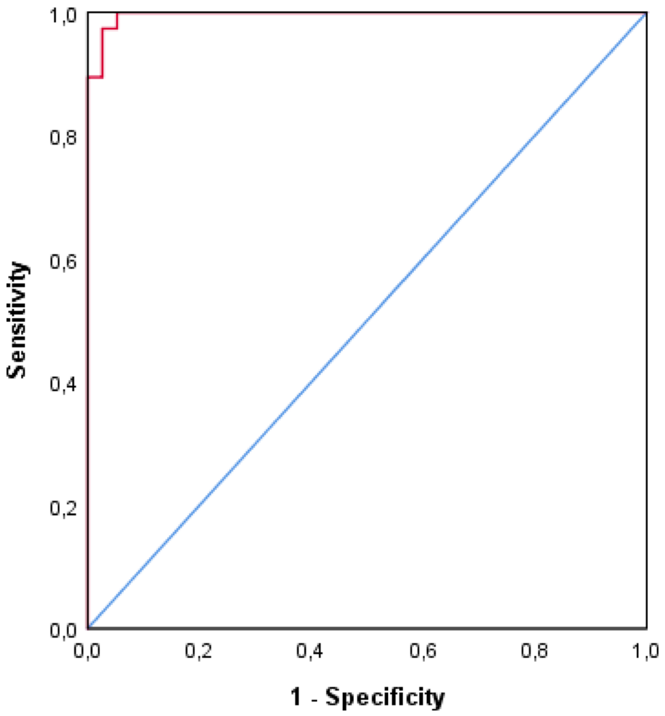

Supplementary Figure 3: ROC analysis based on tumor tissue samples of the GCT patients and the corresponding contralateral tissue (AUC: 0.997).

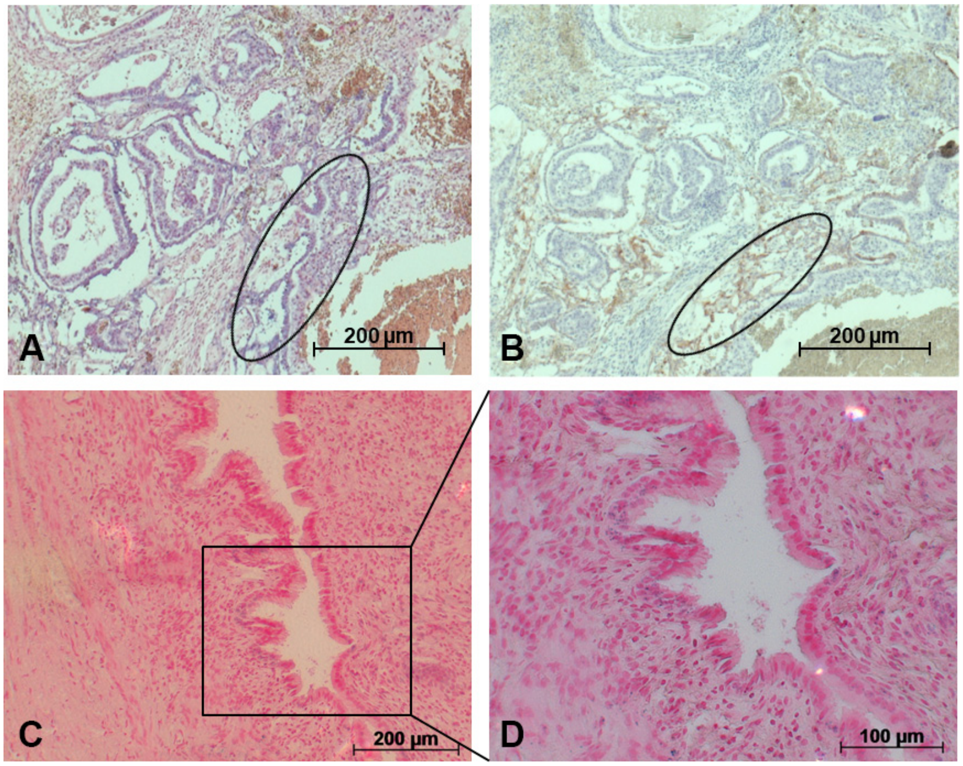

Supplementary Figure 4: Detection of miR-371a-3p in GCT mixed tumor including YST, EC and CH and teratoma via *in situ* hybridization. (**A**) *In situ* hybridization with a probe against miR-371a-3p causes blue staining in cells in a mixed tumor. (**B**) Immunohistochemical staining of the same area with Glypican 3 antibody for identification of YST cells. Circles indicate the same areas of YST cells. (**C**) *In situ* hybridization with a probe against miR-371a-3p in pure teratoma. (**D**) Section from C. No blue staining in teratoma cells.
